# Multivariate Geostatistical Modeling and Risk Analysis of Beach Litter: A Case Study of Playa Blanca Beach, Chile

**DOI:** 10.3390/ijerph17228384

**Published:** 2020-11-12

**Authors:** Mohammad Maleki, Kevin Soria

**Affiliations:** Department of Metallurgical and Mining Engineering, Universidad Católica del Norte, Antofagasta 1270709, Chile; soria.kevin.mp@gmail.com

**Keywords:** beach litter, geostatistical simulation, beach contamination, risk analysis, environmental hazard

## Abstract

Beach litter is a worldwide problem that has several negative effects. A first step in preventing an environmental hazard is to determine and model the level of contamination. In this paper, geostatistical simulation is used to model two main forms of beach litter (cigarette butts and sharp items) in one of the most contaminated beaches in Antofagasta, Chile. A hundred realizations of cigarette butts and broken glass are generated to emulate their joint spatial distribution. The simulation results are used to classify the beach into different areas with respect to the risk of injury by broken glass and the level of contamination by cigarette butts. The models obtained can be used by local authorities in beach clean-up programs and by visitors to beaches in choosing the safest and cleanest areas. The results demonstrate the capability of geostatistical simulation algorithms to model different types of beach litter.

## 1. Introduction

Beach litter is a significant problem worldwide with negative effects in terms of the reduction in environmental biodiversity, human health, and tourism [1,2]. In addition to the risks associated with the leaching of poisonous chemicals [3], beach litter such as broken glass poses the threat of injury to visitors to beaches [2,4,5]. Between 1988 and 1991, 4% of needlestick injuries reported to public health services in the UK occurred at beaches [6]. Investigations regarding Tasmanian beaches found that 21.6% of visitors to even clean beaches were injured by litter [1,2]. Similarly, there were 161, 261 reported cases of beach injuries in New Zealand over a 10 year period (2007–2016) [2].

Generally, the contamination level of beaches is one of the main factors for choosing a beach by visitors. According to a study carried out by Krellin et al. (2017), 85% of people avoid visiting a beach if it has more than 15 pieces of beach litter per m^2^ [7]. Another study found that 97% of surveyed people stated that they would avoid beaches with more than 10 large items of litter per m^2^ [8].

**Figure 1:**
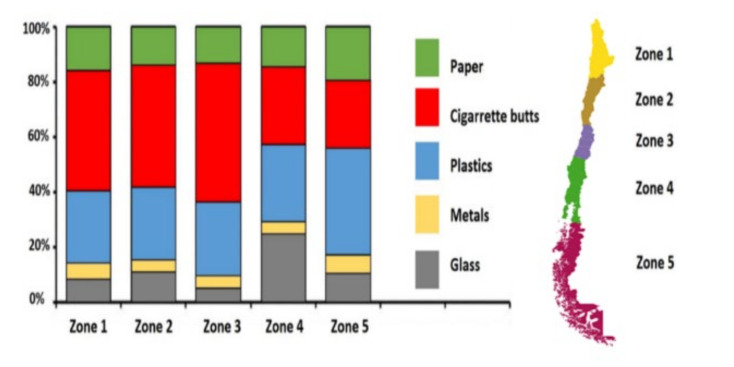
Percentage of different beach litters in five zones of Chile.

The main sources of beach litter are human activities. Unfortunately, in the last two decades, the amount of beach litter has increased considerably, such that contamination of beaches is currently recognized as a worldwide problem [9,10]. Therefore, effective action is crucial to make beaches safer and cleaner for users and to prevent environmental hazards. In the opinion of the authors, the first step to prevent and control beach contamination is to model and determine the level of contamination, which would lay the foundations for policies and administrative decision-making on this issue. Since 2007, an organization in Chile called *cientificos de basura* has been measuring beach litter on the main beaches in cities throughout the country. The group has found that the beaches in Antofagasta, northern Chile, are the most contaminated [11]. Moreover, according to the same study, plastics, cigarette butts and broken glass are the most common contaminants on Chilean beaches. Figure 1 shows the percentage of different beach litters in five different zones of Chile [11].

In this study, cigarette butts and broken glass were modeled by taking advantage of geostatistical methods on one of the most popular beach of Antofagasta. The beach under study receives many visitors during each year and due to human activities, a high level of contamination can be observed in the beach.

## 2. Methodology

Geostatistical tools and algorithms are widely used for modeling regionalized data and solving spatial interpolation problems for both natural and anthropogenic variables [12,13,14,15,16,17,18]. In this context, we decided to apply geostatistical methods to model cigarette butts and broken glass on a beach in Antofagasta, since these two items respectively represent the most common and the most dangerous forms of litter.

As a first step, cigarette butts and broken glass were sampled in the study area. Then, by taking advantage of multivariate geostatistical methods, cigarette butts and broken glass were modeled in the study area. Based on the results, the study area was classified into different sub-areas and the probability of exposure to sharp items (broken glass and bottle caps) was calculated. Finally, the results were processed to assess the capability of geostatistical algorithms to model beach litter.

### 2.1. Study Area

**Figure 2:**
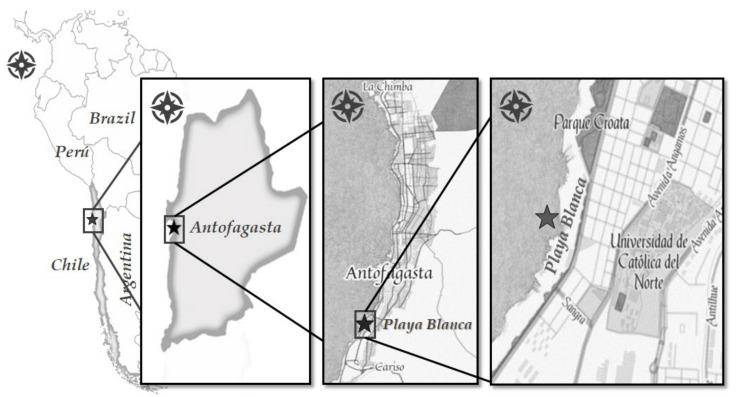
Location of the beach under study.

As noted above, Antofagasta has the most contaminated beaches in Chile [11], which is why we decided to study and model beach litter on one of the beaches in the city. Antofagasta is located in northern Chile and is the capital city of the Antofagasta Region. The chosen beach, called Playa Blanca, is located in the southern part of the city (Figure 2).

Playa Blanca is one of the most popular beaches in Antofagasta because of its proximity to the largest city park (Parque Croata), the two main universities, and many bars and restaurants. The level of contamination and beach litter varies during the year and usually reaches a peak in summer when the number of visitors is the highest. It is important to notice that this beach has no formal cleaning programs. The study area of 50 m × 150 m is part of Playa Blanca beach.

### 2.2. Sampling Methodology

**Figure 3:**
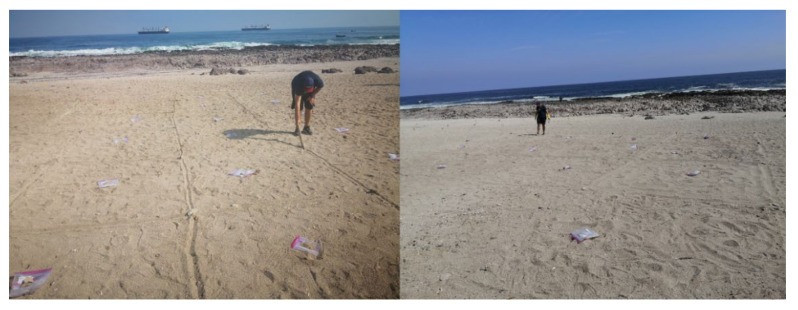
Sample grids.

As a first step, this area was divided into a 1 m by 1 m grid (Figure 3). During the sampling, the authors realized that there is a strong relationship between the presence of cigarette butts and broken glass, resulting from the fact that many people smoke and drink at the same time.

As a result, a high concentration of cigarette butts is expected in an area with a high concentration of broken glass (mainly from beer bottles). Consequently, we applied multivariate geostatistical algorithms to jointly simulate these two forms of beach litter in the study area (Playa Blanca, Antofagasta). During sampling process, metal caps of beer bottles were also found in some grids and as they present the same danger and can cause serious injuries, a new category was generated called ‘sharp items’, which includes broken glass and metal caps. Therefore, hereafter in this study, the term ‘sharp items’ refers to broken glass and metal caps. However, it is worth noting that the sharp items were mostly corresponding to broken glass.

The cigarette butts and sharp items (pieces of broken glass and metal caps) found in each grid were counted. The broken glass was mainly small fragments of beer bottles. Samples were gathered not only on the surface of the study area, but also from approximately 5 cm below the surface. To preserve the same conditions for all samples, the sampling was conducted on one day in January (when beach litter is usually at a maximum) with the help of twenty volunteers. Before starting sampling, the volunteers were trained to reduce the sampling errors. The samples were gathered at a time when there were practically no visitors to the beach.

To apply geostatistical methods, the average densities of cigarette butts and pieces of broken glass per square meter were used, rather than simply the count per grid cell.

### 2.3. Geostatistical Modeling

#### 2.3.1. Variogram Analysis

**Figure 4:**
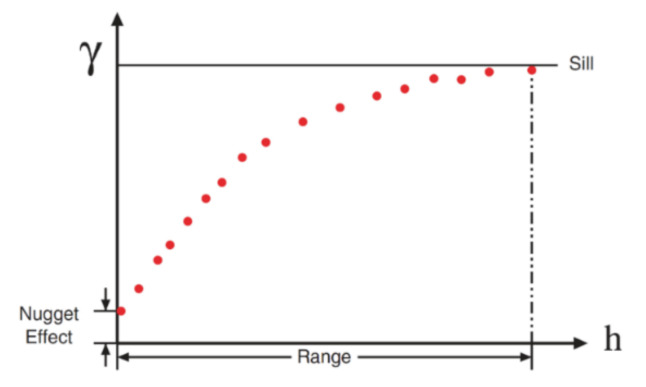
An example of experimental variogram.

In addition to the number and position of sampling data, geostatistical algorithms consider the distance between data and target points and the spatial continuity to model the underlying variables [19]. The experimental variogram is the main tool to determine the spatial continuity of the variable under consideration, which is defined below:

(1) $$\hat{\gamma }\left(h\right)=\frac{1}{2N\left(h\right)}\overset{N\left(h\right)}{\underset{i=1}{\sum }}{\left(z\left(x_{i}+h\right)-z\left(x_{i}\right)\right)}^{2}$$

where $z\left(x_{i}+h\right)$ and $z\left(x_{i}\right)$ are the values observed at points $x_{i}$ and $x_{i}+h$ in space, h is the distance between the two points, and $N\left(h\right)$ is the number of pairs of data separated by h. Figure 4 shows a typical variogram. As in this case, variograms are usually increasing in function. The slope of the variogram determines how rapidly the influence of the sampling data decreases with the distance.

The variogram usually reaches its limits (or sill) at a distance called the range, which indicates the distance from which two data cease to be correlated. The value at very small distances (just beyond the sample size) is called the nugget effect and determines the small-scale variability of the variable.

In the presence of more than one variable, it is necessary to calculate cross-variograms between variables. The experimental cross-variogram determines the dependence between two variables at a given separation distance, and is defined as follows:

(2) $${\hat{\gamma }}_{ab}\left(h\right)=\frac{1}{2N\left(h\right)}\overset{N\left(h\right)}{\underset{i=1}{\sum }}\left(z_{a}\left(x_{i}+h\right)-z_{a}\left(x_{i}\right)\right)\left(z_{b}\left(x_{i}+h\right)-z_{b}\left(x_{i}\right)\right)$$

where $z_{a}\left(x_{i}\right)$ and $z_{b}\left(x_{i}\right)$ are the values of variables a and b observed at location $x_{i}$. When a = b, one finds the variogram as defined in (1), called the direct variogram.

#### 2.3.2. Geostatistical Simulation

Geostatistical simulation is used to provide multiple outcomes (as many as desired), each of which is considered a possible scenario reproducing the spatial continuity of the variable(s) under study. Apart from providing a prediction of the unknown values at unsampled locations, by averaging the scenarios, one of the advantages of geostatistical simulation is to allow users to define the level of uncertainty and to analyze risks [19]. The rationale is to interpret each variable as a realization of a spatial random field and to draw realizations of this very random field over the area of interest. The realizations can be furthermore conditioned to the sampling data, in order that all the realizations match these data.

The turning-band algorithm [20,21] was used in this work to generate one hundred conditional realizations of two underlying variables that were simulated together because of the high correlation between them. This algorithm simplifies the simulation problem in multidimensional spaces to a set of simulations along lines that span the two- or three-dimensional space, while ensuring the reproduction of the desired spatial correlation structure (direct and cross-variograms). As the algorithm produces realizations of Gaussian random fields, the data need to be transformed into Gaussian data (normal scores) prior to variogram analysis and simulation; the simulated Gaussian values are then back-transformed to the original scale.

## 3. Results

### 3.1. Geostatistical Simulation

**Figure 5:**
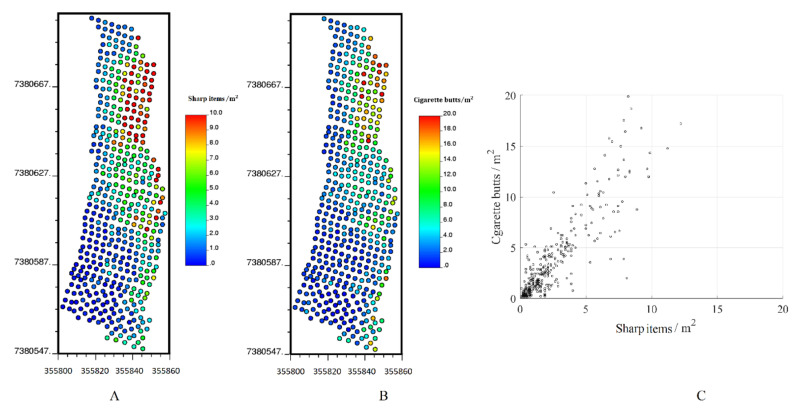
Location maps of sampling data: (**A**) cigarette butts, (**B**) sharp items. (**C**) Scatter plot between cigarette butts and sharp items.

Overall, cigarette butts and sharp items were gathered at 530 sampling locations in the study area (Figure 5). Table 1 shows the main statistics of the collected data. As noted above, there is a high correlation between the two variables, which allowed us to model the two variables jointly.

**Figure 6:**
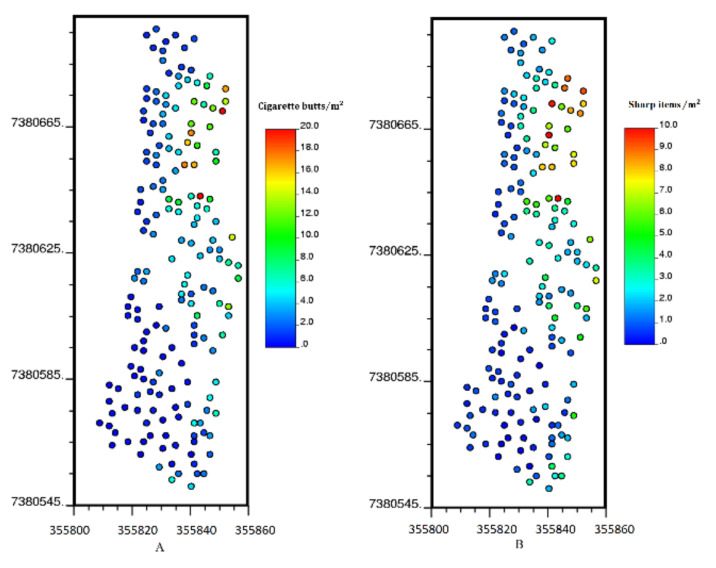
Location maps of training data: (**A**) cigarette butts, (**B**) sharp items.

To assess the capability of the geostatistical approach, the sampling data were divided into two subsets: 200 data were chosen randomly (training set) and used for modeling cigarette butts and sharp items in the study area, while the remaining 320 sampling data (testing set) were used for performing split-sample validation. Figure 6 shows the locations of the 200 training data used for modeling cigarette butts and sharp items.

**Figure 7:**
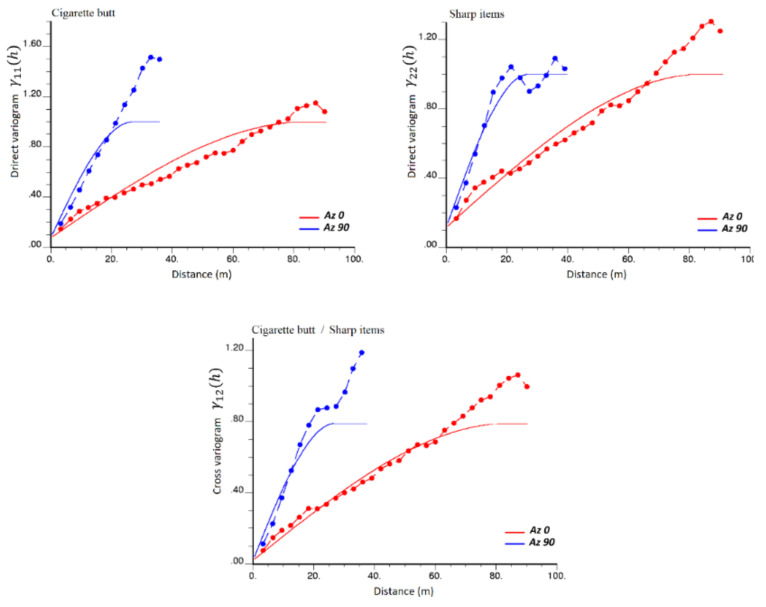
Experimental (dashed lines) and modeled (solid lines) variograms of the normal scores data along two mains anisotropy directions, Az 0° (Red) and Az 90° (Blue). Top: direct variograms. Bottom: cross-variogram.

A joint variogram analysis was applied to the normal score transforms of the training data. Figure 7 and Equation (3) show the direct and cross-variogram models between the two Gaussian variables along the two main anisotropy directions, which includes a nugget effect and a spherical model with range 84 m along the direction of azimuth 0° and 27 m along the direction of azimuth 90°:

(3) $$\left(\begin{array}{cc} \gamma_{11}\left(h\right) & \gamma_{12}\left(h\right)\\ \gamma_{21}\left(h\right) & \gamma_{22}\left(h\right) \end{array}\right)=\left(\begin{array}{cc} 0.075 & 0.02\\ 0.02 & 0.115 \end{array}\right)Nugget\;+\left(\begin{array}{cc} 0.925 & 0.77\\ 0.77 & 0.885 \end{array}\right)Sph\left(84\;m,27\;m\right)$$

The sill matrices associated with the nugget and spherical structures are positive semidefinite, which ensures that the model is mathematically consistent [22]. In Equation (3), index “1” refers to cigarette butts and index “2” to sharp items.

**Figure 8:**
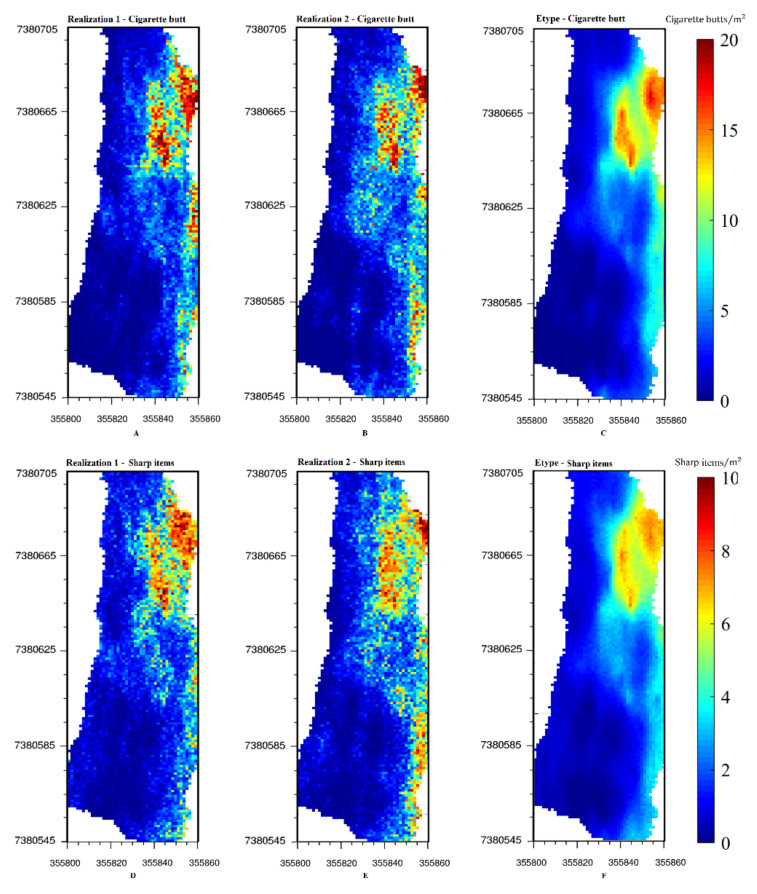
Two generated realizations of (**A**,**B**) cigarettes butt, (**D**,**E**) sharp items. Average of one hundred realizations for (**C**) cigarette butts and (**F**) sharp items.

The two variables were simulated jointly and 100 conditional realizations were generated with the turning-bands algorithm. Figure 8 shows the first two realizations and the average of one hundred realizations for each variable. As can be seen from Figure 8C,F, there is a high concentration of sharp items and cigarette butts in the northeastern part of the study area, which is the most contaminated. Generally, it can be observed that there is less beach litter in the western part, that is, closer to the ocean, which could be because visitors tend not to sit so close to the ocean.

### 3.2. Post Processing of Simulation Results

The results of simulation were used to classify the whole study area according to the risk of injury by sharp items and the level of contamination. To the authors’ knowledge, there are no specific international standards or criteria to classify beaches with respect to sharp items (broken glass or bottle caps) and cigarette butts. Recently, [23] proposed the Hazardous Items Index (HII) for classifying a beach with respect to hazardous items. This index can be calculated considering all amounts of hazardous items or by differentiating between sharp items such as broken glass or toxic items such as cigarette butts. This index determines the possibility of being affected by hazardous item and is calculated as follows [23]:

(4) $$HII=\frac{\frac{\sum hazardous\;marine\;litter}{Log_{10}\sum total\;marine\;litter}}{Total\;Area\;of\;beach\;}\times K$$

with *K* = 20. According to the calculated HII, each beach is classified into one of the following categories from a hazardousness point of view (Table 2).

By using the results of simulation, the HII index was calculated for the study area with respect to the sharp items. On average over 100 realizations, it is found equal to 9.4, which indicates that most of the area is covered by broken glass or bottle cap and there is a high probability of being affected by sharp items in this part of Playa Blanca beach.

The cleanliness of study area also was assessed by using the Clean Coast Index (CCI) presented by Alkalay et al. (2007) for classifying beach cleanliness with respect to the presence of plastics. This index determined the level of cleanliness of beach and is calculated as follow:

(5) $$CCI=\frac{\sum Total\;beach\;litter}{Total\;Area\;of\;beach\;}\times K$$

with *K* = 20. According to the calculated CCI index, the beach will be classified into one the five following categories from cleanliness point of view (Table 3).

In this criterion, all types of beach litter are considered for calculating the Clean Coast Index. Therefore, by using the results of simulation and considering both sharp items (broken glass and bottle cap) and cigarette butt, the CCI index of the study area was calculated. According to calculated index (CCI = 110.8) the study area is classified as ‘Extremely Dirty’.

These two indexes can be calculated globally, for the whole beach, or locally, for different sectors of the beach [23]. However, in addition to the whole study area, we are interested in classifying each simulated grid of the study area. Moreover, almost all sampled sharp items (pieces of broken glass and bottle cap) had a small size, which meant they were not very easy to see at first glance, whereas most studies record only fairly large items, and small items are often not reported [24]. For instance, the smallest gathered sample in the study carried out by Alkalay et al. (2007) was 2 cm long. Therefore, we decided to establish two criteria for classifying each simulated node of the study area by using the simulation results. The studied area was divided into four classifications according to the number of cigarette butts per square meter:

- Very Clean: $0\leq \mathrm{cigarette}\;\mathrm{butts}/m^{2}<1$
- Clean: $1\leq \mathrm{cigarette}\;\mathrm{butts}/m^{2}<2$
- Dirty: $2\leq \mathrm{cigarette}\;\mathrm{butts}/m^{2}<6$
- Very Dirty: $6\leq \mathrm{cigarette}\;\mathrm{butts}/m^{2}$

The studied area was also divided into the three groups based on the risk of injury by sharp items:

- Low risk of injury: $0\leq \mathrm{Sharp}\;\mathrm{items}/m^{2}<1$
- Moderate risk of injury: 1 $\leq \mathrm{Sharp}\;\mathrm{items}/m^{2}<3$
- High risk of injury: $3\leq \mathrm{Sharp}\;\mathrm{items}/m^{2}$

**Figure 9:**
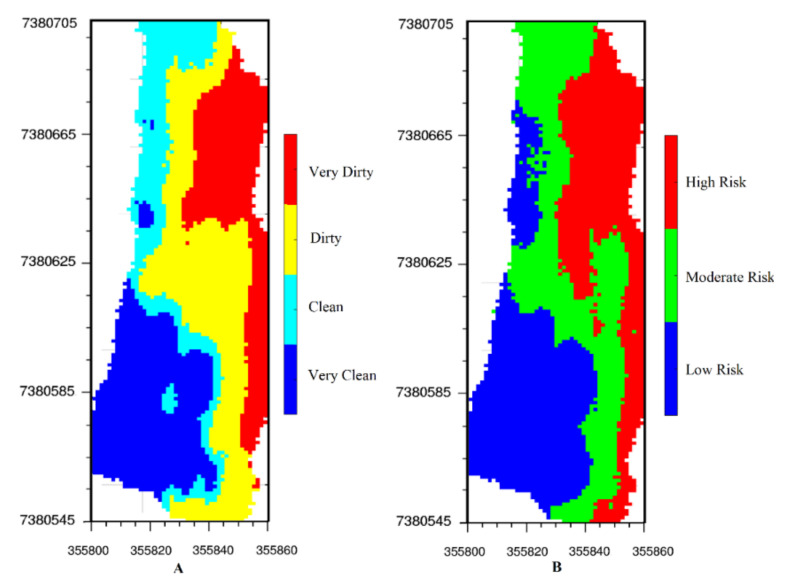
Classifying the beach according to (**A**) the level of contamination by cigarette butts (**B**) the risk of exposure to the sharp items.

Figure 9 shows the classification results. Most of the beach is considered very dirty and there is a high risk of injury due to the presence of broken glass and bottle caps.

**Figure 10:**
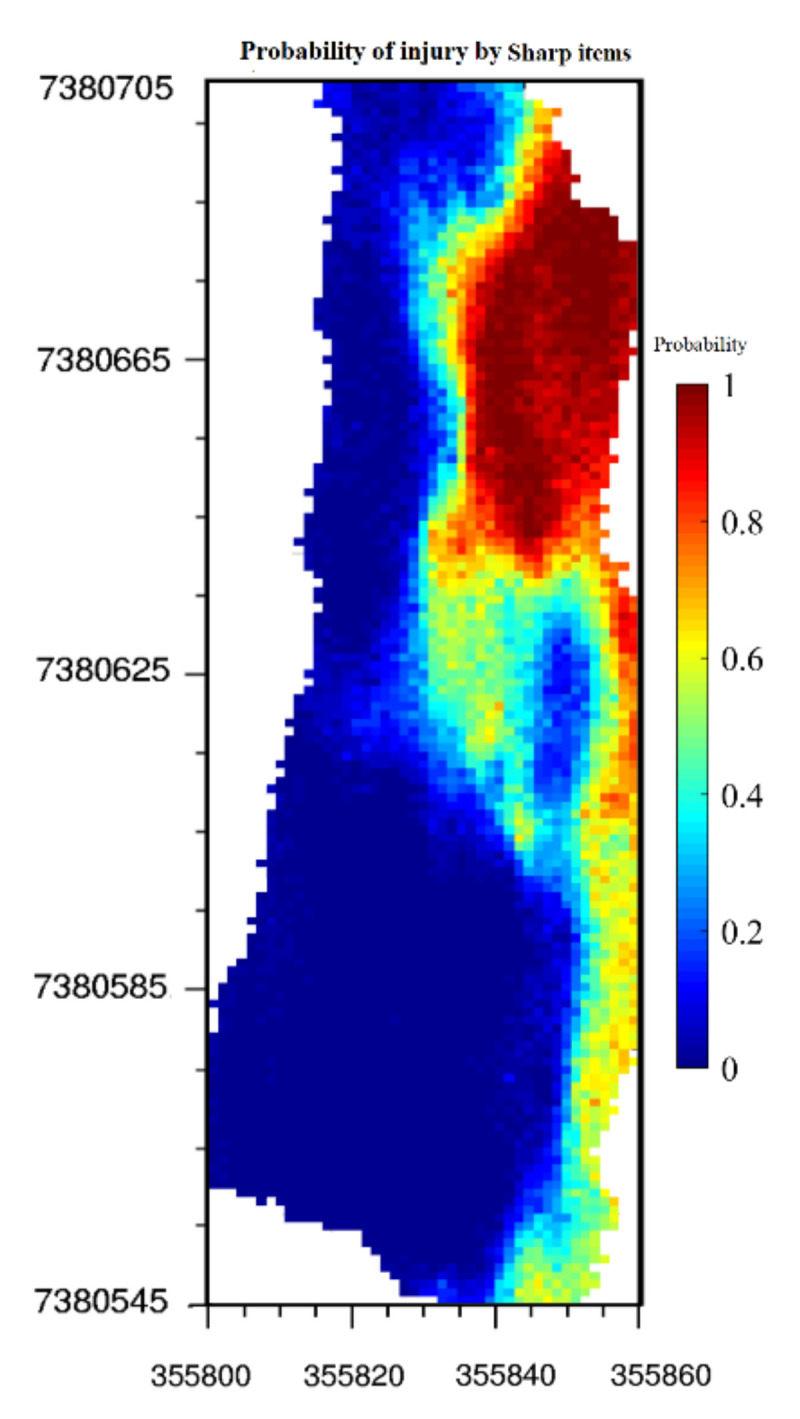
Map of the probability of injury by sharp items calculated on the basis of hundred generated realizations.

Because each generated realization is a possible scenario for the variables, the risk can be analyzed and probability maps of the variables can be constructed. The probability that a variable exceeds a specific threshold in any given grid node can be calculated based on one hundred realizations. Figure 10 shows the probability of injury by sharp items in the study area (probability of more than three pieces of broken glass or metal caps being in a grid node). For instance, the probability of injury by sharp items in a specific grid node is 80% if, from one hundred simulated values for this grid node, eighty of them are equal to or more than 3. As can be observed from Figure 10, the probability of injury is generally higher in the eastern part than in the western part, which is expected as there is a high concentration of sharp items in the eastern part of the study area.

### 3.3. Validation of Results

Split-sample validation can be used to determine the accuracy and quality of the simulated values. As previously mentioned, the original sampling data (530 samples) were divided into two subsets, via a random selection. Then, one of the subsets (training subset including 200 sampling data) was used as conditioning data for simulating the cigarette butts and sharp items at the locations of the other subset (testing subset). The simulated values at the testing subset, as well as their average, can finally be compared to the true values.

**Figure 11:**
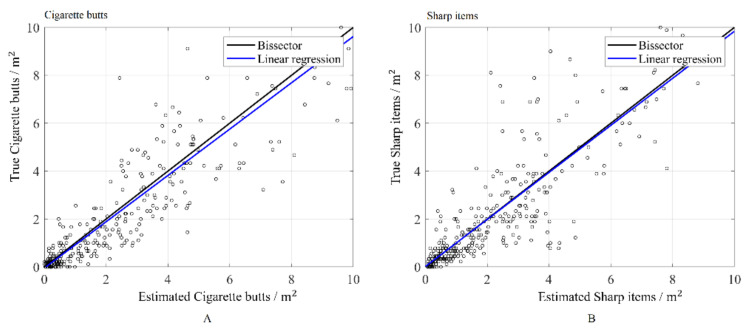
Split–sample validation of (**A**) cigarette butts (**B**) sharp items.

**Figure 12:**
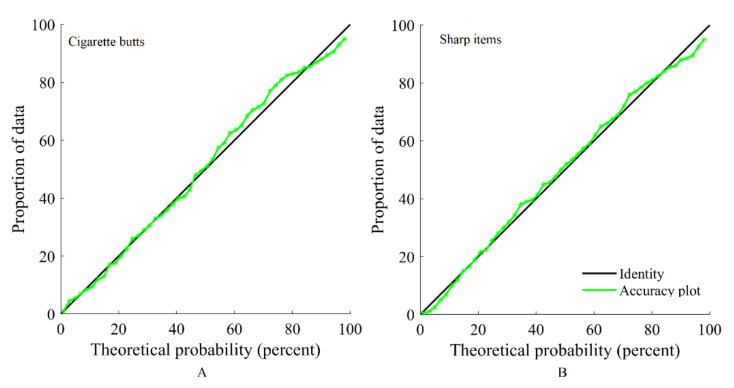
Accuracy plots for (**A**) cigarette butts, and (**B**) sharp items.

As observed in Figure 11, the results of this process suggest that the proposed approach is accurate for both cigarette butts and sharp items, insofar as the regression of the true values upon the estimated values (average of 100 realizations) is close to the identity. The results can also be validated with a graph termed an accuracy plot [25]. The realizations are used to obtain a local distribution of the variables at each location of the testing subset. Then, an interval can be constructed considering a given probability p. This process is repeated many times for different probabilities. One then draws the theoretical probability versus the observed proportion of data for which the true value falls into the probability interval. The results of the simulation are accurate if the points are close to the diagonal, as is the case here for both variables (Figure 12).

## 4. Discussion

The calculated CCI and HII indexes determine that the study area is extremely dirty and there is a high probability of being affected by sharp items (broken glass and bottle cap) in this part of the beach. As demonstrated in Figure 8C,F, the northeastern part of the study area is the most contaminated, which can be explained because it receives more visitors owing to the presence of benches and the fact that it offers more shade than other parts of the beach, making it more comfortable for sitting and for recreational activities. Despite this fact, visitors still tend to use this part of beach because they are unaware of the risk given that the pieces of broken glass are small and cannot be seen easily. This demonstrates the usefulness of the beach litter model since it can warn visitors of the risks posed by contaminated areas of the beach. These maps have different uses.

For example, these maps can be useful for beach cleaning programs, which are usually conducted by regional governments. In addition, the visitors can use them to choose the best place to sit in terms of presenting the least contamination and the lowest risk of injury. These maps can be used to monitor and manage the phases of environmental restoration.

In this study, sharp items and cigarette butts were modeled in a small part of a beach to demonstrate the capacity of geostatistical modeling. However, different types of beach litter, such as plastic, paper, and small pieces of metal, on other beaches can be also modeled. In addition, other type of contamination such as chemical pollution which can be present in the beaches can be modeled by using geostatistical methods.

It is worth noting that the beach litter models vary with time and cannot be generalized for the entire year. For instance, the level of contaminations usually peaks in summer as more people visit beaches. Therefore, samplings should be taken for every season.

The sampling was one of the main challenges in this works as it directly affected the obtained models of beach litter. Therefore, in order to preserve the same conditions for all measurements, the sampling was carried out over the course of one day and the volunteers were trained in order to reduce sampling errors. Another point to consider is the size of the pieces of broken glass. In this study, almost all sampled pieces of broken glass had the same small size. However, in a situation where the pieces were of different sizes, the larger pieces should be considered as they pose more danger. In this case, the authors recommend weighing the broken glass in each grid node instead of counting the number of pieces.

## 5. Conclusions

Beach litter is a multidimensional problem with negative effects on the biodiversity of habitats and species, human health and tourism. Beach cleanliness is, in fact, the most important factor people consider in relation to visiting a beach [8]. Beach litter is also a real threat to human health [1]. Using geostatistical algorithms, this study simulated cigarette butts and sharp items (broken glass and bottle caps) as respectively the most common and most dangerous forms of litter on one of the most contaminated beaches in Antofagasta. Based on the simulation results, CCI and HII indexes were calculated for the whole study area, indicating cleanliness and the probability of being affected by sharp items, respectively. The results demonstrate that the study area is extremely dirty and also there is a very high probability of being affected by sharps items. The high contamination of the study area would be due to the lack of cleanliness programs and implies the urgent need for effective actions for cleaning the beach.

In addition to the whole study area, each simulated node of the study area was also classified according to the risk of injury by sharp items and the level of contamination by cigarette butts. To this end, simple criteria were established to define the thresholds for every category. In addition, one hundred simulated realizations were used to calculate the map of probability of injury by broken glass. The results can be useful for visitors to beaches in choosing the cleanest and safest parts of a beach. The results can also be used by local authorities in undertaking beach cleanliness programs. In summary, in this paper, we tried to demonstrate the capability and versatility of geostatistical simulation algorithms to model beach litter and to analyze the risk of injury.

Finally, it can be deduced that geostatistical approaches are helpful tools that can be applied in environmental science for modeling different contaminated material, such as beach litter. One advantage of these approaches is their applicability when one has an incomplete survey and a few sampling data. Even in this case, one can estimate the contaminated material at unsampled locations with high accuracy.

## Figures and Tables

**Table 1:** Main statistics of sampling data.

	Cigarette Butts/m^2^	Sharp Items/m^2^
Number of samples	530	530
Mean	3.57	2.39
Standard deviation	4.0	2.4
Minimum	0.11	0.11
Lower quartile	0.77	0.66
Median	1.94	1.55
Upper quartile	4.67	2.56
Maximum	24.11	12.22
Correlation coefficient	0.87	

**Table 2:** Hazardous Items Index (HII) classification.

HII	Category	Description
0	I	No hazardous beach litter is seen
0.1–1	II	Some hazardous beach litter is seen over a large area.
1.1–4	III	A considerable amount of hazardous beach litter is seen.
4.1–8	IV	A lot of hazardous beach litter items are on the beach.
+8	V	Most of the area is covered by hazardous beach litter

**Table 3:** Clean Coast Index (CCI) classification.

CCI	Category	Description
0–2	Very Clean	No beach litter is seen
2.1–5	Clean	No beach litter is seen over a large area
5.1–10	Moderate	Items of beach litter can be detected
10.1–20	Dirty	A lot of beach litter items are on the beach
+20	Extremely Dirty	Most of the area is covered by beach litter.
